# From On-Target to Off-Target Activity: Identification and Optimisation of *Trypanosoma brucei* GSK3 Inhibitors and Their Characterisation as Anti-*Trypanosoma brucei* Drug Discovery Lead Molecules

**DOI:** 10.1002/cmdc.201300072

**Published:** 2013-06-14

**Authors:** Andrew Woodland, Raffaella Grimaldi, Torsten Luksch, Laura A T Cleghorn, Kayode K Ojo, Wesley C Van Voorhis, Ruth Brenk, Julie A Frearson, Ian H Gilbert, Paul G Wyatt

**Affiliations:** [a] Drug Discovery Unit (DDU), Division of Biological Chemistry and Drug Discovery, College of Life Sciences, University of DundeeSir James Black Centre, DD1 5EH (UK) E-mail: i.h.gilbert@dundee.ac.ukp.g.wyatt@dundee.ac.uk; [b] Division of Allergy and Infectious Diseases, Department of Medicine, University of WashingtonSeattle, WA 98195 (USA)

**Keywords:** antiprotozoal agents, GSK3, medicinal chemistry, protein kinases, *Trypanosoma brucei*

## Abstract

Human African trypanosomiasis (HAT) is a life-threatening disease with approximately 30 000–40 000 new cases each year. *Trypanosoma brucei* protein kinase GSK3 short (*Tb*GSK3) is required for parasite growth and survival. Herein we report a screen of a focused kinase library against *T. brucei* GSK3. From this we identified a series of several highly ligand-efficient *Tb*GSK3 inhibitors. Following the hit validation process, we optimised a series of diaminothiazoles, identifying low-nanomolar inhibitors of *Tb*GSK3 that are potent in vitro inhibitors of *T. brucei* proliferation. We show that the *Tb*GSK3 pharmacophore overlaps with that of one or more additional molecular targets.

## Introduction

Human African trypanosomiasis (HAT), also known as African sleeping sickness, is a parasitic disease caused by protozoan parasites of the species *Trypanosoma brucei* and is fatal if untreated. HAT is endemic in certain regions of sub-Saharan Africa, with around 50 million people at risk of infection across 25 countries. The number of reported cases of HAT has fallen recently and is now at about 10 000 reported new cases per year; however, the actual number of cases is estimated to be much higher (30 000–40 000 new cases per year).^1^–^3^

Following infection by the bite of a tsetse fly, patients initially suffer from phase 1 disease, in which they experience episodes of fever, headache, sweating, and swelling of the lymph nodes. Phase 2 disease results from the spread of infection into the central nervous system (CNS). Patients begin to experience a disturbance in their circadian rhythm, resulting in bouts of fatigue alternating with manic periods, which progress to daytime slumber and nighttime insomnia, with progressive mental deterioration leading to coma and death. Generally the disease is diagnosed only when it has already progressed to the phase 2 CNS stage.

HAT is a neglected disease, because despite millions of people being under the threat of infection, there is no commercial market to justify funding drug development. There are only two stand-alone drugs available for the treatment of late-stage sleeping sickness: melarsoprol and eflornithine. However, both drugs have serious limitations such as toxicity, complex parenteral administration, which is poorly suited to a rural African setting, low and variable brain penetration, the development of resistant parasites,^4^ and patient compliance.^5^ A combination therapy of nifurtimox and eflornithine was recently approved for the treatment of stage 2 HAT primarily due to a cost benefit and improved convenience of the new treatment over eflornithine alone. Unfortunately, resistance to nifurtimox develops rapidly in the laboratory.^6^–^8^

In recent years a number of drug development initiatives funded by foundations and/or governments have begun to address the need for improved drugs to treat stage 2 HAT.^9^ Two new oral clinical candidates were recently developed: fexinidazole,^10^ a nitroimidazole derivative that is currently in clinical development, and SCYX-7158,^11^ a benzoxaborole derivative that has been selected for entry into clinical development. However, owing to the high rates of attrition in drug discovery and the requirement for multiple drugs to combat the development of resistant parasites, the pipeline must be further enhanced.

There is a lack of validated drug discovery targets and lead compounds for HAT and other neglected diseases.^12^ Protein kinases have been explored as possible targets for HAT, as they play important roles in virtually every cellular event from cell division to stress response.^13^ Kinases are druggable targets, and crystal structures have been published for many of them.^14^ Bioinformatics searches of the *T. brucei* genome identified 176 parasite protein kinases,^15^, ^16^ making this family an attractive source of novel drug discovery targets for the treatment of HAT and other parasitic diseases.^17^–^19^

Human GSK3β (*Hs*GSK3β) is involved in the regulation of a vast array of cellular processes in eukaryotes: insulin signalling, growth factors, nutrient levels, cell fates during embryonic development, cell division, apoptosis, and microtubule function.^20^ *Hs*GSK3β has been investigated as a drug target for many diseases, from diabetes to neurodegenerative diseases. To aid development, the crystal structure of *Hs*GSK3β has been solved, and high-affinity small-molecule inhibitors of *Hs*GSK3β have been developed.^14^, ^21^–^24^ Whilst the precise role of the trypanosome orthologue GSK3 short kinase (*Tb*GSK3) in the bloodstream form of *T. brucei* has yet to be determined in terms of parasite biology, the importance of this enzyme has been demonstrated by RNA interference experiments that showed decreased growth rates for parasites in in vitro culture.^25^, ^26^

Herein we report our studies on the identification and optimisation of *Tb*GSK3 inhibitors with potent antiparasitic activity and highlight their potential for the development of new therapies for the treatment of HAT.

## Results and Discussion

### Homology modelling

**Figure 1:**
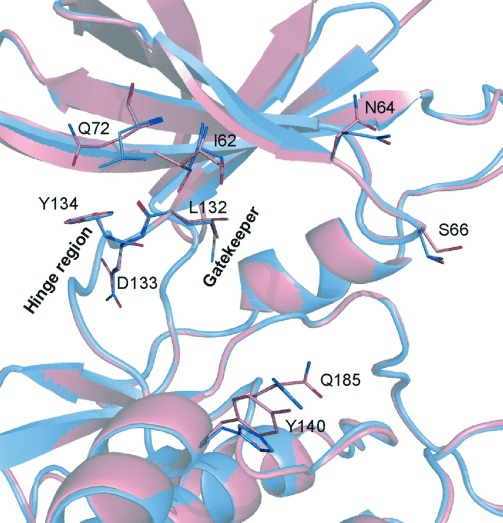
Superposition of the *Hs*GSK3β crystal structure (PDB code 1R0E) with the homology model of *Tb*GSK3. The human structure is shown in pink, the *Tb*GSK3 homology model in blue; both structures are shown in ribbon representation. Binding pocket residues of *Tb*GSK3 that differ from those of *Hs*GSK3β are represented as sticks. Regions of the kinase site are labelled according to previously published conventions.^28^

Crystal structures of *Leishmania major*^27^ and human GSK3 have been published. The tertiary structure of *Lm*GSK3 (PDB code 3E3P) is very similar to that of *Hs*GSK3β (PDB code 1R0E), but several binding pocket residues are missing in the *Leishmania* crystal structure, as no clearly defined electron density was present. In addition, no ligand is bound in the *Lm*GSK3 crystal structure. Therefore, we selected the crystal structure of *Hs*GSK3β as the template to build a homology model for *Tb*GSK3. The *Tb*GSK3 sequence is 52 % identical and 71 % similar to the sequence in the *Hs*GSK3β structure (PDB code 1R0E). Hence *Hs*GSK3β provides a template for 91 % of the *Tb*GSK3 sequence (amino acids 20–348) which allowed a reliable model to be built (Figure 1). Analysis of the ATP binding pockets revealed amino acid differences that could be exploited to design selective inhibitors (Table 1).

**Table 1:** Residue differences in the ATP binding sites of *Hs*GSK3β and *Tb*GSK3

Kinase pocket region	*Hs*GSK3β^[a]^	*Tb*GSK3^[b]^
Hinge region	D133	E
	Y134	F
Gatekeeper	L132	M
E1	Y140	H
	Q185	H
Adenine pocket	I62	A
	Q72	L
G-loop	N64	Q
	S66	T

[a] PDB code 1R0E. [b] Homology model.

### Hit discovery

**Figure 2:**
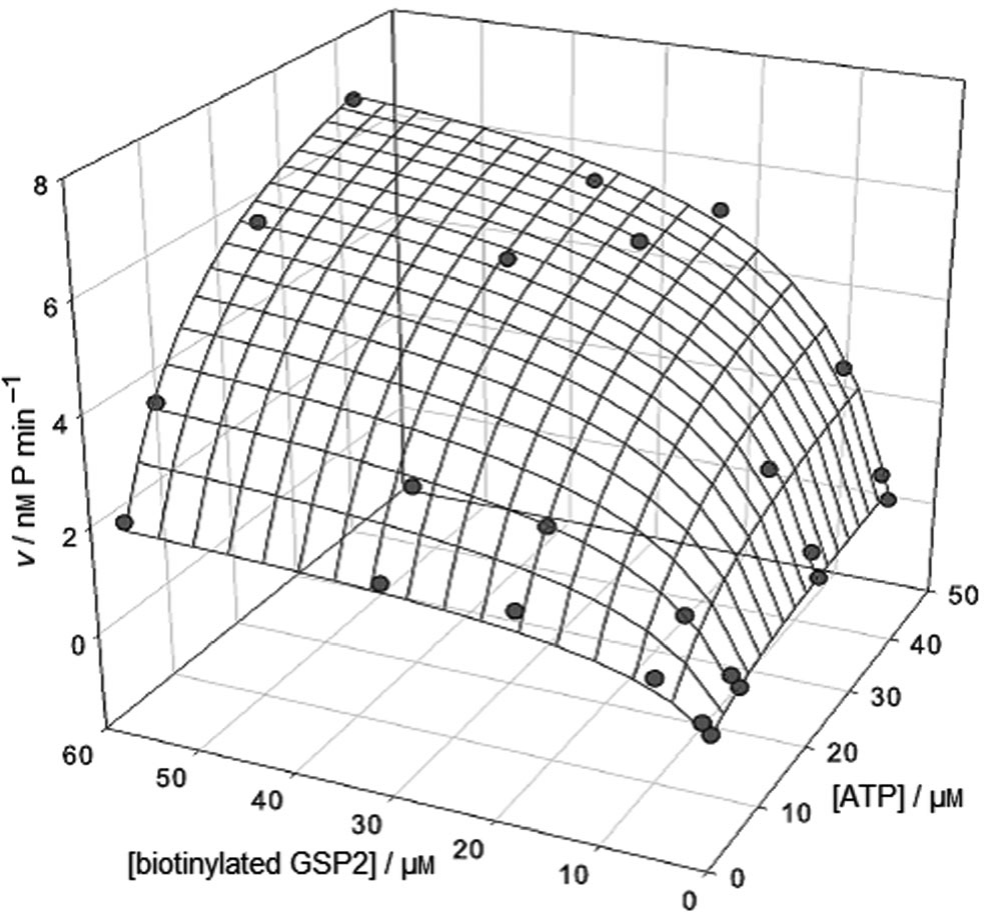
Determination of kinetic parameters for *Tb*GSK3. *K*_M_ values for the ATP and GSP2 substrates were determined in a time-course matrix experiment in which the initial velocities (*v*) were determined as product formed (P, nm) per minute. The grid represents the best fit obtained by globally fitting the data to the equation for the random-order rapid equilibrium model with cooperation parameter (*α*) equal to 1.

Recombinant *Tb*GSK3 was produced as previously described.^25^ The kinetic parameters were determined by measuring initial reaction velocities in a matrix experiment of varied ATP and peptide substrate concentrations. The *K*_M_ value of *Tb*GSK3 for the substrate with sequence YRRAAVPPSPSLSAHSSPHQ[pS]EDEEE (GSP2) and ATP were 8.4±1.3 and 11.0±1.8 μm, respectively, with no evidence of cooperativity (Figure 2). The determined *K*_M_ values for GSP2 and ATP are similar to those previously reported of 2.4 and 4.5 μm, respectively.^25^

**Figure 3:**
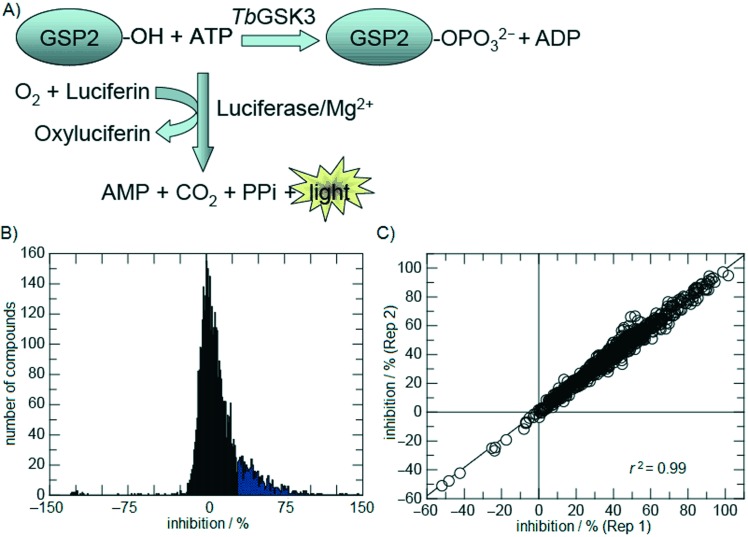
Format and results of the primary screen. A) A KinaseGlo assay format was adopted for the primary screen based on luminescence detection. B) Distribution of the percent inhibition of the focused kinase set. Hits were selected by setting 3 standard deviation units from the average of high controls as threshold (≥30 % inhibition). C) 567 selected hits were retested in duplicate, and the two replicate values showed high correlation.

For the primary screen, a 384-well KinaseGlo (Promega) luminescence-based assay was used as previously described.^25^ In this assay, luminescence is inversely related to kinase activity and directly related to ATP depletion (Figure 3 A). GW8510 was used as a standard inhibitor^6^ (figure S1, Supporting Information). The DDU kinase set of 4110 compounds^29^ was screened in single point at 25 μm providing robust data (*Z*′=0.61±0.06; Table 2). From this, 567 compounds with a percentage of inhibition >30 % were retested in a duplicate-point screen, to give 517 reconfirmed compounds with inhibition values ≥30 % (12.8 % of the library; Figure 3 B,C).

**Table 2:** Assay conditions and screening statistics

	KinaseGlo	Flashplate
*Tb*GSK3	7.5 nm	2.5 nm
GSP2	3.2 μm	1 μm
ATP	1 μm (<*K*_M_)	1 μm (<*K*_M_)
*Z*′	0.61±0.06	0.80±0.08
GW8510 IC_50_	10±0.2 nm	6±4 nm

**Figure 4:**
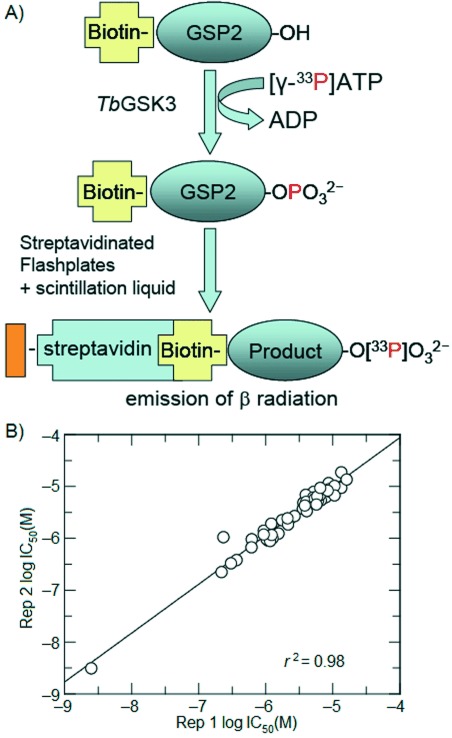
Format and results of the potency test. A) A flashplate radiometric assay format was adopted for the potency determination based on selective capture of the biotinylated phosphorylated product by the streptavidin-coated plates. B) The 100 most potent hits were tested in 10-data point curves in two independent determinations; the correlation plot between the log IC_50_ values of the replicates is reported.

For hit validation and all subsequent compound potency determinations, a radiometric 96-well Flashplate assay (PerkinElmer) was adopted (Figure 4 A). Although the KinaseGlo format has many advantages for screening of chemical libraries, its reliance upon ATP consumption means it requires a level of substrate consumption >10 % to achieve an acceptable signal window; it is therefore unsuitable for accurate IC_50_ determinations. The Flashplate assay was performed at an ATP concentration below the enzyme′s *K*_M_ value for ATP, so that the *K*_i_^app^ approximated the measured IC_50_ value, aiding the assessment of selectivity. Potency evaluation (10-point curves) was carried out in duplicate for the 100 most potent compounds. As in the primary assay, the potency assay format yielded highly robust data (*Z*′=0.80±0.08; Table 2). The compounds exhibited a range of potencies for *Tb*GSK3 which were highly reproducible, with an *r*^2^ value of 0.99 for two replicates (Figure 4 B), with 15 compounds having IC_50_ values <1 μm. The identity and purity of hits taken into potency determination were confirmed by LC–MS.

### Prioritisation of hits and series definition

**Figure 5:**
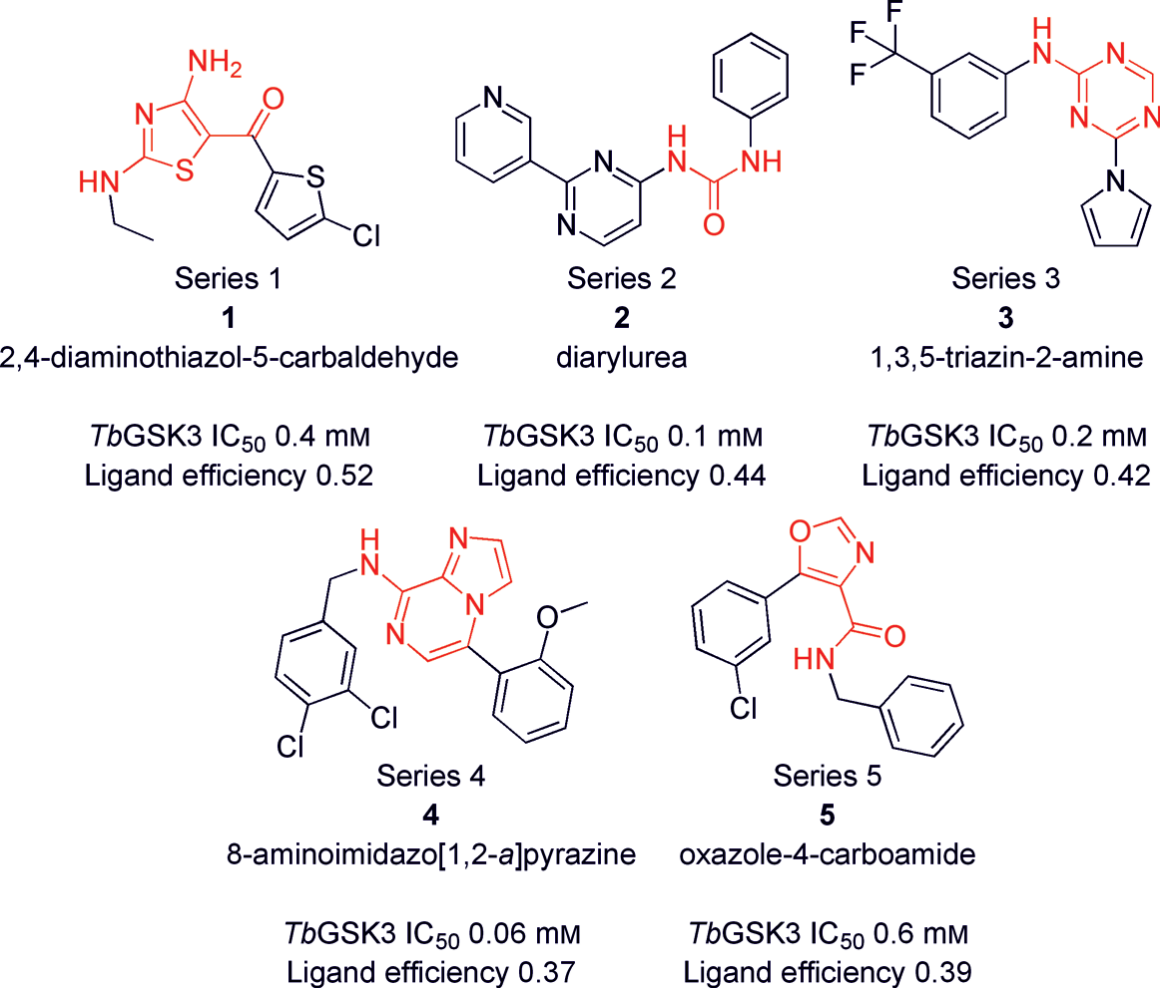
Series classification. Common features representative of each series are highlighted in red. Ligand efficiencies are given as kcal mol^−1^ per heavy atom.

To prioritise the hits, hit compounds were rank ordered by their ligand efficiencies against *Tb*GSK3, in which ligand efficiency=[−*RT* ln(IC_50_)]/*N*_non-H atoms_.^30^ Subsequently, the highest-priority compounds were grouped into series based on structural similarity (Figure 5), resulting in a number of different compound series. Several of the *Tb*GSK3 inhibitors identified have ligand efficiencies >0.4 kcal mol^−1^ per heavy atom, beyond the commonly used guideline of 0.3 kcal mol^−1^ per heavy atom, which relates to an optimised lead with an IC_50_ value of 10 nm and 38 non-hydrogen atoms (*M*_r_∼500 Da).^30^ In particular, diaminothiazole **1** is a highly ligand-efficient starting point, with a *Tb*GSK3 ligand efficiency of 0.52 kcal mol^−1^ per heavy atom.

### Hit validation

Five promising hit series containing ligands with ligand efficiencies >0.3 kcal mol^−1^ per heavy atom were progressed into hit series validation. Series 2 was based around a diaryl urea and exemplified by compound **2**. Compounds of this series were also identified as broad-spectrum kinase inhibitors with toxicity toward mammalian cell lines during our previously published investigations of the CRK3 kinase, so no further work was conducted with this compound series.^31^

Series 3 was based around 2-amino-1,3,5-triazines; 36 examples of this series were contained in the screening library with compound **3** being the only active example (Figure 5). Due to the limited commercial availability for representatives of this series and the apparent requirement for specific substituents, this series was not pursued further.

The 8-aminoimidazo[1,2-*a*]pyrazines (series 4) were also identified in a previous DDU project and were found to be broad-spectrum kinase inhibitors that are toxic to human cell lines (MRC5 cells). In addition, the lead compound **4** has an unfavourable calculated log *P* value of 4.8 (Figure 5). Therefore, no further work was carried out on this series.

Eleven oxazole-4-carboxamides (series 5) were identified in the high-throughput screen (HTS), with compound **5** inhibiting *Tb*GSK3 at a sub-micromolar IC_50_ value (Figure 5). Over 900 examples of this compound series were commercially available at the time of our study, and more than 100 compounds were selected for purchasing and testing. However, none of these compounds demonstrated activity in the *T. brucei* cell assay. This, combined with the relatively poor *Tb*GSK3 IC_50_ value of 0.1 μm observed within the series after testing more than 140 examples, as well as the flat SAR, it was decided that this series would not be pursued any further.

Compound **1** (series 1) was the only 2,4-diaminothiazol-5-carbaldehyde present in the DDU kinase screening set at the time of screening (Figure 5). Although **1** was a singleton, it has good predicted physical properties (*M*_r_=288 Da, log *P*=2.1, TPSA=68 Å^2^), a ligand efficiency of 0.52 kcal mol^−1^ per heavy atom for *Tb*GSK3, and demonstrated promising activity in a *T. brucei* proliferation assay (EC_50_ 2 μm). Of slight concern is the presence of a ketone functionality, which has the potential to interact with nucleophiles within the cell; this would have to be monitored during compound development. Based on these considerations, it was decided to progress this compound to hit validation. As a side note, compound **1** is also a very effective *Hs*GSK3 inhibitor, with an IC_50_ value of 5 nm (*n*=2) and an outstanding ligand efficiency of 0.67 kcal mol^−1^ per heavy atom. Thus, **1** may also be an excellent starting point for a human GSK3 drug discovery programme.

### Structure-guided design

**Figure 6:**
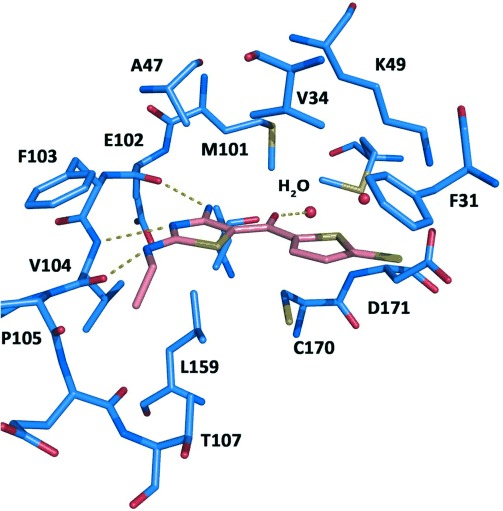
Proposed binding mode for **1** in the homology model of *Tb*GSK3. Both, ligand and protein are represented in sticks and colour-coded by atom type. Ligand carbon atoms are shown in salmon, and protein carbon atoms in light blue. Putative hydrogen bonds to the ligand are shown as yellow dotted lines (other hydrogen bonds within the active are not shown for clarity).

A potential binding mode for compound **1** in the homology model of *Tb*GSK3 was generated using Moloc (Gerber Molecular Design, Switzerland; Figure 6). In the proposed binding mode the 2,4-diaminothiazole moiety forms three hydrogen bonds with the protein backbone of the *Tb*GSK3 hinge (Glu102, Phe103, and Val104). Furthermore, the thiazole group is sandwiched between Leu159 and Ala47 and undergoes hydrophobic interactions with both amino acids. In addition, the ligand carbonyl group is proposed to be in plane with the core thiazole and forms an intramolecular hydrogen bond with the primary amino group. A water molecule, observed in many *Hs*GSK3β structures, was kept for the binding mode generation and is predicted to mediate a hydrogen bond between the inhibitor carbonyl lone pair and the backbone amide NH group of Asp171. The presence of an analogous water molecule in the recently solved X-ray crystal structure of *Lm*GSK3 (PDB code 3E3P) gives further evidence that the water molecule is conserved and plays an important role in GSK3–ligand binding. The 2-chlorothiophene moiety is predicted to form lipophilic interactions with Phe31 and Cys170, while the ethyl group points toward Leu159, and the hydrophobic part of Thr107 potentially contributes van der Waals interactions to the binding affinity.

Docking was carried out in order to guide the hit expansion, with the aim of confirming the aminothiazoles as a series of *Tb*GSK3 inhibitors, improving in vitro potency, and to build SAR. A series of 112 commercially available 2,4-diaminothiazoles with various substituents were docked into the homology model of *Tb*GSK3 using FlexX.^32^ The docking solutions were visually inspected, and 21 compounds were selected for purchase (Table 3).

**Table 3:** Activity of key compounds from series 1

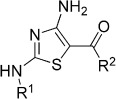			IC_50_ [μm]		EC_50_ [μm]	
Compd	R^1^	R^2^	*Tb*GSK3	*Hs*GSK3β	*T. brucei*	MRC5
**1**	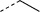	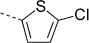	0.4	0.005	2	>15
**6**	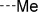	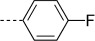	25	ND^[a]^	7	>50
**7**	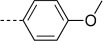	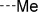	1	ND^[a]^	13	25
**8**	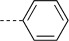	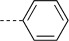	0.03	0.007	0.2	13
**9**	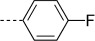	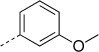	0.05	0.003	0.1	>50
**10**	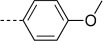	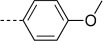	0.1	0.007	0.7	4

[a] Not determined.

### Biological results and structure–activity relationships

The biological data for early hit optimisation are summarised in Table 3. The hit compound **1** has an ethyl group at R^1^ which may form hydrophobic interactions with Leu159 and the hydrophobic part of Thr107 (Figure 6, Table 1). Although no direct analogues were available, the closely related methyl analogue **6** is around 50-fold less active than **1**, suggesting that lipophilic bulk in the R^1^ region is beneficial. Introducing an aromatic group into R^1^ and truncating R^2^ to methyl was tolerated, with an IC_50_ value of 1 μm for **7**. Increasing the size of R^1^ while maintaining a lipophilic group at R^2^ gave a 10-fold improvement in activity, with several examples demonstrating IC_50_ values of <100 nm for *Tb*GSK3, such as compounds **8**, **9**, and **10**.

In general the 2,4-diaminothiazol-5-carbaldehydes tested were found to be more potent inhibitors of *Hs*GSK3β than they are of *Tb*GSK3, confirming that selectivity between *Hs* and *Tb*GSK3 can be achieved, albeit initially in the undesired direction. In contrast, in cellular assays, the lead molecules are selective antiparasitic agents as exemplified by **9**, which has an EC_50_ value of 0.13 μm against *T. brucei*, but no activity against MRC5 cells (EC_50_>50 μm).

**Figure 7:**
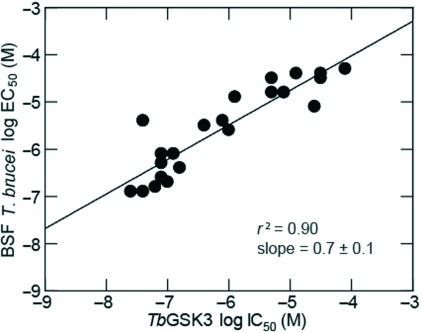
Correlation between inhibition of *Tb*GSK3 and inhibition of *T. brucei* cell growth for the initial set of compounds. Supporting Information table S1 lists the compounds used to derive the correlation plots along with the *Tb*GSK3 log IC_50_ and *T. brucei* log EC_50_ values.

A correlation plot of cell efficacy (bloodstream form (BSF) *T. brucei* log EC_50_) against enzyme potency (*Tb*GSK3 log IC_50_) gave a strong correlation for the early members of this series (correlation coefficient: 0.90) with a 5-fold drop off between the *Tb*GSK3 and *T. brucei* activities (Figure 7 and Supporting Information table S1). Considering that the physiological level of ATP in *T. brucei* is in the millimolar range, whilst in our *Tb*GSK3 potency assay, the concentration was 1 μm, we expected the IC_50_ value for *Tb*GSK3 in cells to drop off by at least 100-fold according to the Cheng–Prusoff equation [Eq. (1)].^33^ In addition, other factors such as protein binding or the requirement for a high level of enzyme inhibition to achieve a phenotypic effect, as observed previously for other *T. brucei* targets, could even result in a >100-fold drop off.^34^ The much lower observed difference between IC_50_ and EC_50_ suggested that the mode of action of series 1 may not be just through inhibition of *Tb*GSK3.


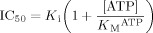
(1)

The small difference between potency against the enzyme and the cell activity for this series led us to consider that there may be more than one mechanism of action driving the cell activity. Substituted 2,4-diaminothiazoles have been described, and examples are known to be potent inhibitors of *Hs*GSK3β,^35^ several CDKs,^36^, ^37^ p25,^37^ and PDE4B.^38^ Interestingly, there are also reports of a 2,4-diaminothiazole (DAT1) which binds to and disrupts microtubules.^39^ Homologues of these targets are present in *T. brucei* and could therefore be modulated by compounds of this series. Additionally, prolific kinase inhibitors often show toxicity toward cells in culture. Compound **8** was profiled at 10 μm against the mammalian protein kinase panel at the University of Dundee, which at the time of testing consisted of 76 mammalian kinases. In agreement with our biochemical assays, compound **8** potently inhibited *Hs*GSK3β; it also inhibited CDK2, MKK1, ERK8, and HIPK2 by >90 % at this concentration (Supporting Information table S2). The human protein kinase profile was sufficiently clean for an early-stage kinase inhibitor project, and we decided to monitor the kinase profile as the series was developed.

### Hit-to-lead development

**Scheme 1:**
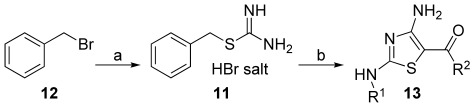
*Reagents and conditions*: a) thiourea (1 equiv), EtOAc, 120 °C, 5 min, b) isothiocyanate (1.1 equiv), DIPEA (1.2 equiv), DMF, RT, 24 h, then 2-bromoketone (1.2 equiv), DIPEA (2.1 equiv), RT, 1 h.

Based on the promising data obtained for the series we decided to progress the project into hit-to-lead development. A stepwise solid-supported two-step synthetic route to diaminothiazoles has been published, starting from the non-commercial reagent, benzyl carbamimidothioate hydrobromide **11**.^40^ We modified the synthesis so that both steps are carried out in a one-pot, solution-phase reaction (Scheme 1). Benzyl bromide **12** was treated with thiourea to give benzyl carbamimidothioate hydrobromide **11** in 89 % yield. Benzyl carbamimidothioate **11** was allowed to react with an isothiocyanate in the presence of Hünig′s base, 2-bromoketones were then added along with an additional equivalent of Hünig′s base to give the desired 2,4-diaminothiazoles **13** in a three-component one-pot synthesis with yields ranging from 3 to 69 %.

The most potent compounds identified in the early work were 2,4-diamino-5-ketothiazoles **13** bearing aromatic groups in both the R^1^ and R^2^ position, such as compound **8** (IC_50_=0.03 μm, Table 3). Although we had identified several *Tb*GSK3 inhibitors with IC_50_ values less than 1 μm, we wished to improve the selectivity over *Hs*GSK3β. Our design explored R^1^ and R^2^ groups of varying size, shape, and polarity to probe the limits of the ATP binding pockets of the *Tb*GSK3 and *Hs*GSK3β enzymes to identify regions where selectivity could be achieved (Table 4).

**Table 4:** Activity of key compounds from series 1

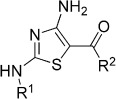			IC_50_ [μm]		EC_50_ [μm]	
Compd	R^1^	R^2^	*Tb*GSK3	*Hs*GSK3β	*T. brucei*	MRC5
**8**	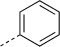	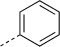	0.03	0.007	0.2	13
**14**	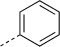	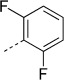	0.02	0.005	0.2	1.6
**15**	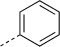	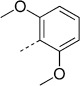	0.4	2	0.4	20
**16**	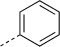	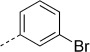	0.1	0.005	0.2	>50
**17**	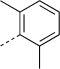	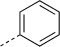	0.2	0.2	9	35
**18**	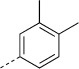	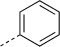	0.06	0.002	0.2	14
**19**	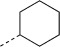	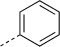	0.2	0.002	0.1	11
**20**	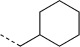	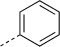	0.7	0.02	0.4	41
**21**	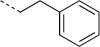	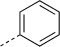	0.5	0.003	6	38
**22**	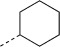	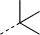	12	ND^[a]^	0.3	>50
**23**	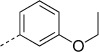	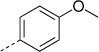	0.04	0.007	4.1	0.3

[a] Not determined.

Initial work focused on the R^2^ group. Introduction of *ortho* substituents, which would be expected to twist the R^2^ group out of plane with the ketothiazole core, was well tolerated for small groups such the fluorine-substituted analogue **14** (IC_50_=0.02 μm). The larger and more electron-rich 2,6-dimethoxyphenyl **15** (IC_50_=0.4 μm) was over 10-fold less active than **14** against *Tb*GSK3. Lipophilic bulk in the *meta* position of the R^2^ substituent was tolerated, but provided no significant potency gains, with 3-bromo analogue **16** (IC_50_=0.1 μm) showing similar activity to the phenyl analogue **8** (IC_50_=0.03 μm).

The R^1^ substituent was then investigated. The 2,6-dimethylphenyl derivative **17** (IC_50_=0.2 μm) is approximately 10-fold less active against *Tb*GSK3 than **8** (IC_50_=0.03 μm). Increasing the *meta* and *para* lipophilic bulk at R^1^ had no benefit, with 3,4-dimethylphenyl **18** similar to **8**. Replacing the R^1^ phenyl ring with cyclohexyl **19** (IC_50_=0.19 μm) caused a small (sixfold) decrease in potency against the enzyme. Insertion of one or two methylene units between the amine and the cyclohexyl and aromatic groups gave small decreases in activity against *Tb*GSK (**20**, IC_50_=0.7 μm; **21**, IC_50_=0.5 μm). As part of our strategy to test the limits of the *Tb*GSK3 and *Hs*GSK3β pockets, the tetrahedral lipophilic *tert*-butyl ketone **22** was synthesised. This compound is ∼300-fold less active than **8** toward *Tb*GSK3; however, it is a potent inhibitor of *T. brucei* growth in vitro, with an EC_50_ value of 0.3 μm, and is also highly selective (>150-fold) over the human MRC5 cell line.

### Biological characterisation of 2,4-diaminothiazol-5-carbaldehydes

**Figure 8:**
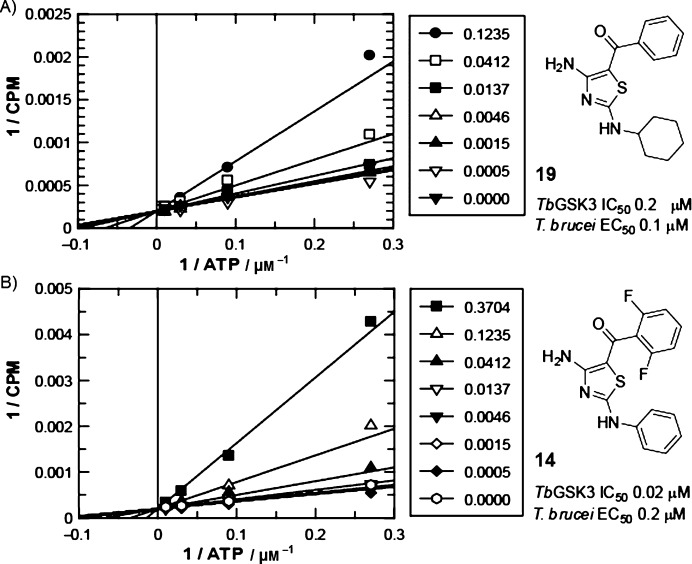
Mechanism of inhibition was determined for A) **19** and B) **14**. Rates (CPM) were determined at the reported inhibitor concentrations (μm) with four varied concentrations of ATP at saturating concentration of the other substrate. The resulting Lineweaver–Burk plots were examined for diagnostic patterns for competitive inhibition and globally fitted to the equation for competitive inhibition.

Studies of the mechanism of inhibition by compounds from the diaminothiazole series (**19** and **14**) confirmed that they are ATP-competitive inhibitors of *Tb*GSK3, as evident from the Lineweaver–Burk plots (Figure 8). The calculated *K*_i_^app^ values (0.25±0.03 and 0.05±0.01 μm for **19** and **14**, respectively) correlated very well with the determined IC_50_ values in the biochemical assay (Table 4) as expected, considering that the level of ATP in the assay was below the *K*_M_ value for ATP.

**Figure 9:**
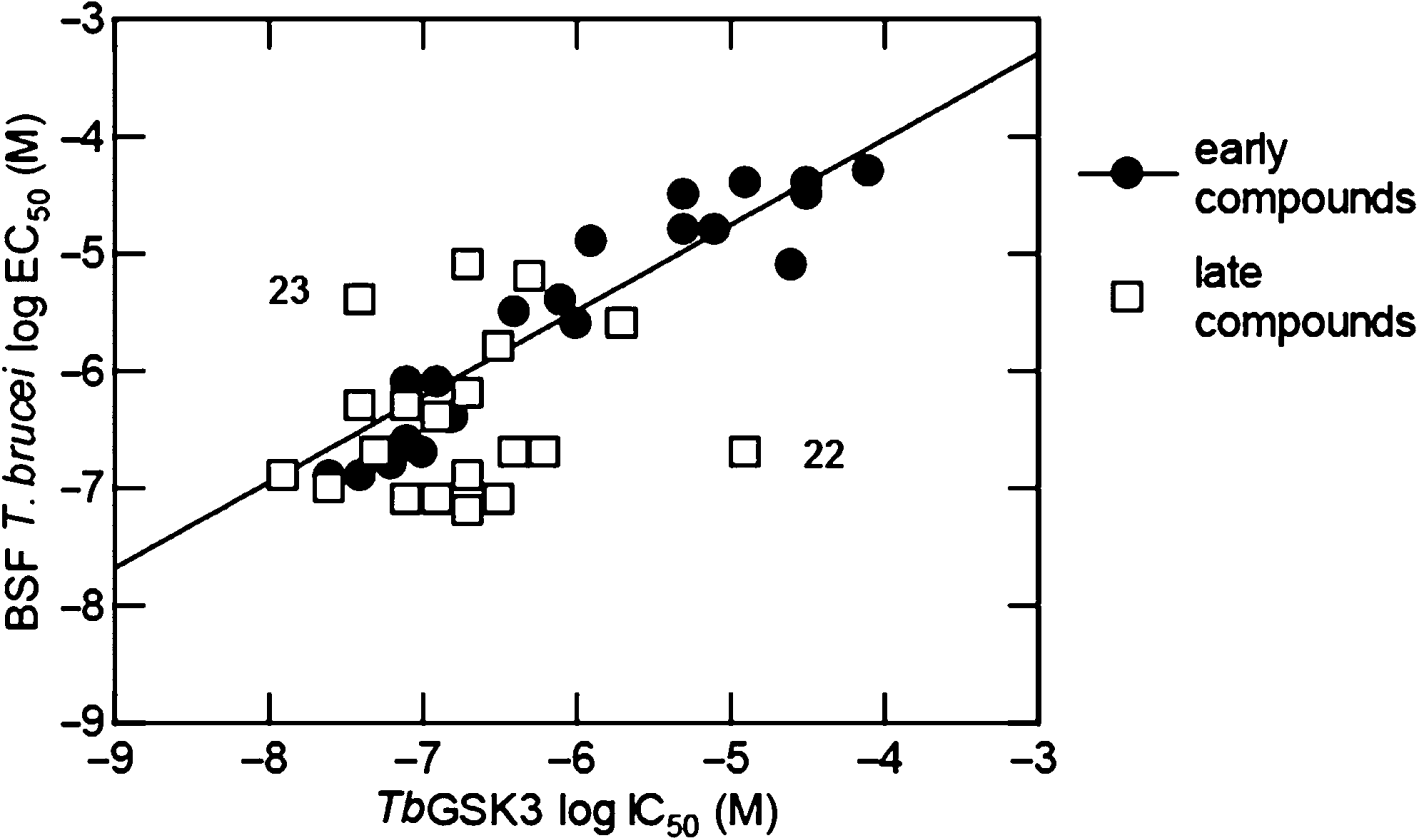
Correlation between inhibition of *Tb*GSK3 and inhibition of *T. brucei* cell growth for the initial set of compounds. The later compounds show weaker correlation. Supporting Information table S1 lists the compounds used to derive the correlation plots along with the *Tb*GSK3 log IC_50_ and *T. brucei* log EC_50_ values. Figure 9 was generated using both the early and later examples of series 1.

During analysis of the SAR it became apparent that the inhibition of cellular (*T. brucei*) growth was no longer tracking with inhibition of the enzyme, *Tb*GSK3. This was particularly apparent when a correlation plot of *T. brucei* log EC_50_ values against *Tb*GSK3 log IC_50_ values was produced for all of the compounds generated by this stage in the programme (Figure 9 and Supporting Information table S1). At the extremes of this plot, the ratio of *T. brucei* EC_50_ values/*Tb*GSK3 IC_50_ values for **23** is 115, whilst for **22** it is 0.017, a difference of ∼6700-fold (Table 4). We believe that the reason for this variation is that an essential molecular target or targets is present in the *T. brucei* parasite which can be modulated by compounds with a similar pharmacophore to that required for *Tb*GSK3 inhibition.

Earlier in the project we had demonstrated that **8** was not a prolific kinase inhibitor (Table 3 and Supporting Information table S2). To determine whether **22** is a broad-spectrum inhibitor of protein kinases we tested **22** along with **14** and **19** in the University of Dundee kinase panel at a concentration of 10 μm (Supporting Information table S2). Most members of series 1 showed >90 % inhibition against several of the kinases tested. Interestingly, **22** was the least broad-spectrum kinase inhibitor tested and it did not inhibit any kinase at the >90 % level; it only inhibited CDK2 and GSK3 at >70 %. These encouraging data demonstrate that **22** selectively modulates the activity of the unknown parasitic target(s) over a broad range of mammalian kinases, and that these targets can be modulated by drug-like molecules. However, owing to the complex pharmacology of these compounds, and as it is not clear what the off-targets are, the only option will be to optimise these compounds phenotypically, rather than through protein screening.

## Conclusions

In summary, we have shown that a screen of a focused kinase library led to the identification of ligand-efficient inhibitors of *Tb*GSK3 with sub-micromolar potency. The chemotypes we identified have physicochemical properties consistent with the development of orally bioavailable compounds. For the most active series based on 2,4-diaminothiazoles, the compounds were also active against the parasite *T. brucei*. Some of the examples had potencies in the order of 100 nm against the parasite, which represents a very good starting point for a drug discovery programme against HAT. However, it quickly became apparent that this series has one or more molecular targets in addition to *Tb*GSK3, which contributes strongly to the trypanocidal activity of the compounds. Interestingly, although based on a kinase scaffold, this series had a reasonably clean profile against the 79 mammalian kinases investigated. In the absence of a clear understanding of which molecular target(s) are responsible for anti-trypanosomal activity, the only way to optimise this compound is phenotypically. We shall report results for this in due course.

## Experimental Section

**Homology modelling**: Sequence alignments between *Tb*GSK3β (Tb927.10.13780 in GeneDB) and *Hs*GSK3β were generated with ClustalW.^41^ Subsequently, Modeller 9.2^42^ was used to build homology models of *Tb*GSK3 short, whereas the human GSK3β crystal structure (PDB code 1R0E) served as template.^43^ Modeller was run with default settings, and only the highest-scoring structure was used for further analysis and modelling. The quality of the model was assessed with QMEAN.^44^, ^45^ A total QMEAN score of 0.726 and *Z*-score of −0.50 were calculated, indicating that the model is of good quality.

**Generation of putative binding modes**: An initial binding mode for compound **1** was generated by manually docking the ligand into the ATP binding pocket of the *Tb*GSK3 homology model with the requirement to establish hydrogen bonds to the hinge residues using Moloc. Its position was subsequently optimised with the MAB force field as implemented in Moloc.^46^ To allow for uncertainties in the homology model, the amino acid side chains facing the binding site were kept flexible during minimisation. For the hit expansion, ligands were docked into the active site of the homology model using FlexX.^32^ A highly conserved water molecule (H_2_0 82 in 1R0E) was kept in the structure used for docking. For the active site determination, a radius of 13 Å around the ligand bound in 1R0E was selected, always using complete amino acids. Ligand conformations were calculated using CORINA.^47^ Compounds were docked using default settings.

***Tb*****GSK3 biochemical characterisation**: *Tb*GSK3 short protein with an N-terminal maltose binding protein fusion was cloned, expressed, and purified as previously reported.^25^ For biochemical characterisation of *Tb*GSK3, the kinase assay buffer (25 mm Tris⋅HCl pH 7.5, 10 mm MgCl_2_, 5 mm DTT, 0.02 % CHAPS, 2 U mL^−1^ heparin) and mix of ATP/[γ-^33^P]ATP and GSP2 substrate (YRRAAVPPSPSLSAHSSPHQ[pS]EDEEE; Pepceuticals) were used in a radiometric format (filterplate assay). The *K*_M_ values for the two substrates (ATP and GSP2) were determined by varying the concentrations of both substrates in a time-course matrix experiment.

**Compound library selection** was described previously.^29^

**Primary screen assays**: For the primary screening of the focused kinase inhibitor library, a 384-well KinaseGlo (Promega) luminescence-based assay was used as previously described.^25^ The reactions contained 7.5 nm *Tb*GSK3, 3.2 μm substrate peptide (Pepceuticals), 1 μm ATP, and 25 μm test inhibitor compound in optimised kinase assay buffer. DMSO and an eight-point titration of GW8510 (Sigma) from 1 μm to 50 pm were included as negative and positive controls. Reactions were incubated at room temperature for 1 h and stopped by the addition of KinaseGlo reagent. Plates were then sealed, and the signal was left to stabilise for 1 h in the dark before luminescence was determined using a TopCount NXT HTS counter (PerkinElmer).

**Potency screen assays**: For hit validation and all subsequent compound potency determinations, a radiometric 96-well Flashplate assay (PerkinElmer) was adopted. Compounds were solubilised in DMSO at a top concentration of 3 mm and serially diluted to achieve 10-point titration of final assay concentrations from 30 μm to 0.3 nm with a final DMSO concentration of 1 % (*v*/*v*). The reaction mixtures contained 1 μm biotinylated GSP2 substrate, 1 μm ATP, 3.7 KBq [γ-^33^P]ATP per well, and 2.5 nm *Tb*GSK3 in the *Tb*GSK3 kinase assay buffer. GSK3 inhibitors were screened for selectivity assessment against *Hs*GSK3β as well. For the *Hs*GSK3β assay the reaction mixes contained 1 μm biotinylated GSP2 substrate, 2 μm ATP, 7.4 KBq [γ-^33^P]ATP per well, and 15 nm *Hs*GSK3β in the *Tb*GSK3 kinase assay buffer (25 mm Tris⋅HCl pH 7.5, 10 mm MgCl_2_, 5 mm DTT, 0.02 % CHAPS, 2 U mL^−1^ heparin).

**Mammalian kinase profiling**: Selected compounds were screened against a panel of mammalian kinases routinely run by the Division of Signal Transduction Therapy (DSTT) at the University of Dundee in duplicate at 10 μm. Enzymes included in the panel and assay conditions are reported.^48^ All biochemical assays were carried out below the *K*_M_^app^ value for ATP for each enzyme, allowing comparison of inhibition across the panel.

**Trypanosome and MRC5 proliferation assay**: Measurement of inhibition of the proliferation of MRC5 (human lung fibroblast) cells and *T. brucei* bloodstream-stage cells was performed using a modification of a cell viability assay previously described.^49^ Compounds (50 μm to 0.5 nm) were incubated with 2×10^3^ cells per well in 0.2 mL of the appropriate culture medium (MEM with 10 % foetal bovine serum for MRC-5 cells) in clear 96-well plates. Plates were incubated at 37 °C in the presence of 5 % CO_2_ for 69 h. Resazurin was then added to a final concentration of 50 μm, and plates were incubated as above for a further 4 h before being read on a BioTek flx800 fluorescent plate reader.

**Data analysis**: Determination of the *Tb*GSK3 kinetic parameters was carried out as described previously.^50^ IC_50_ values were determined using a four-parameter equation in XLFit 4.2. To establish mode of inhibition, rates were determined at 10 inhibitor concentrations with four varied concentrations of ATP in saturating concentration of GSP2. The resulting Lineweaver–Burk plots were examined for diagnostic patterns for competitive, mixed, or uncompetitive inhibition. Graphs and analyses were carried out using Grafit 6.0. The correlation between in vitro IC_50_ against *Tb*GSK3 in *T. brucei* cells and inhibitor potency (*K*_i_) for ATP-competitive inhibitors was determined according to the Cheng–Prusoff equation [Eq. (1)].

### Chemistry

^1^H NMR spectra were recorded on a Bruker Avance DPX 500 instrument unless otherwise stated. Chemical shifts (*δ*) are expressed in ppm. Signal splitting patterns are described as singlet (s), broad singlet (br s), doublet (d), triplet (t), quartet (q), multiplet (m), or combination thereof. Low-resolution electrospray (ES) mass spectra were recorded on a Bruker MicroTof mass spectrometer, run in positive ion mode, using either MeOH, MeOH/H_2_O (95:5), or H_2_O/CH_3_CN (1:1)+0.1 % formic acid as the mobile phase. High-resolution electrospray MS measurements were performed on a Bruker MicroTof mass spectrometer. LC–MS analyses were performed with an Agilent HPLC 1100 (Phenomenex Gemini Column 5 m C_18_ 110A 50×3.0 mm, eluting with 20 % MeOH/H_2_O, 0–3 min) and a diode array detector in series with a Bruker MicroTof mass spectrometer. Column chromatography was performed using RediSep 4 or 12 g silica pre-packed columns.

The following compounds were purchased from commercial suppliers and their purity and identity confirmed by LC–MS analysis (all compounds were >85 % pure based on a diode array detector). Compounds **1** and **2** were purchased from ChemBridge Corporation; **3** was purchased from Maybridge; **4** was purchased from BioFocus; **5** was purchased from ChemDiv Inc.; **6**, **9**, and **23** were purchased from TimTec; **7** and **10** were purchased from Specs.

**Benzyl carbamimidothioate hydrobromide (11)**: Benzyl bromide (16.0 mL, 135 mmol), thiourea (10.0 g, 131 mmol), and EtOAc (75 mL) were combined, and then heated at 120 °C in a microwave reactor for 5 min. The reaction was allowed to cool to room temperature, and the resulting solid was collected by filtration to give benzyl carbamimidothioate hydrobromide **11** as a white solid (29.3 g, 118.6 mmol, 89 % yield); ^1^H NMR (500 MHz, [D_6_]DMSO): *δ*=9.07 (br s, 4 H), 7.44–7.32 (m, 5 H), 4.49 ppm (s, 2 H).

**4-Amino-2-(phenylamino)thiazol-5-yl)(phenyl)methanone (8)**: **General procedure A** (used for the synthesis of compounds **8**, **14**–**22**): Benzyl carbamimidothioate hydrobromide (100 mg, 0.4 mmol), phenyl isothiocyanate (52 μL, 0.43 mmol), and *N*,*N*-diisopropylethylamine (DIPEA; 76 μL, 0.43 mmol) were added to DMF (5 mL), and the resulting mixture was stirred at room temperature for 24 h. 2-Bromoacetophenone (96 mg, 0.48 mmol), DIPEA (139 μL, 0.80 mmol), and DMF (2 mL) were then added, and the mixture was stirred at room temperature for 1 h, after which the reaction was quenched by the addition of aqueous HCl (1 m, 4 mL). The product was extracted with EtOAc (3×3 mL), and the combined extracts were back washed with LiCl (2×3 mL of a 5 % *w*/*w* solution in H_2_O), brine (3 mL), and then dried with MgSO_4_, and the solvent was removed under reduced pressure. Purification by column chromatography on silica eluting with petroleum ether (PE) 40–60 °C and EtOAc gave **8** as a yellow solid (47 mg, 0.15 mmol, 39 % yield); ^1^H NMR (500 MHz, [D_6_]DMSO): *δ*=10.80 (br s, 1 H), 8.22 (br s, 2 H), 7.69–7.67 (m, 2 H), 7.62 (d, *J*=7.7 Hz, 2 H), 7.51–7.46 (m, 3 H), 7.39–7.36 (m, 2 H), 7.09 ppm (tt, *J*=7.4 and 1 H*z*, 1 H); ^13^C NMR (125 MHz, DMSO): *δ*=182.7, 167.2, 165.6, 141.9, 139.5, 130.4, 129.1, 128.4, 126.7, 123.4, 119.0, and 92.2 ppm; LC–MS *m*/*z*=296 [*M*+H]^+^, *t*_R_=4.28 min, purity 88 % (Agilent, 20–90 % CH_3_CN, ES+ on an acidic method).

**(4-Amino-2-(phenylamino)thiazol-5-yl)(2,6-difluorophenyl)methanone (14)**: General procedure A gave **14** as a yellow solid (32 mg, 0.10 mmol, 10 % yield); ^1^H NMR (500 MHz, [D_6_]DMSO): *δ*=10.89 (br s, 1 H), 8.31 (br s, 2 H), 8.08 (br s, 1 H), 7.57–7.50 (m, 3 H), 7.39–7.35 (m, 2 H), 7.23–7.19 (m, 2 H), 7.13–7.10 ppm (m, 1 H); LC–MS *m*/*z*=332 [*M*+H]^+^, *t*_R_=4.25 min, purity 90 % (Agilent, 20–90 % CH_3_CN, ES+ on an acidic method).

**(4-Amino-2-(phenylamino)thiazol-5-yl)(2,6-dimethoxyphenyl)methanone (15)**: General procedure A gave **16** as a yellow solid (9 mg, 0.03 mmol, 3 % yield); ^1^H NMR (500 MHz, [D_6_]DMSO): *δ*=10.58 (br s, 1 H), 7.79 (s, 2 H), 7.54 (d, *J*=8.0 Hz, 2 H), 7.54–7.52 (m, 3 H), 7.06 (t, *J*=7.3 Hz, 1 H), 6.69 (d, *J*=8.4, 2 H), 3.71 ppm (6 H, s); LC–MS *m*/*z*=356 [*M*+H]^+^, *t*_R_=4.10 min, purity 100 % (Agilent, 20–90 % CH_3_CN, ES+ on an acidic method).

**(4-Amino-2-(phenylamino)thiazol-5-yl)(3-bromophenyl)methanone (16)**: General procedure A gave **16** as a yellow solid (211 mg, 0.56 mmol, 69 % yield); ^1^H NMR (500 MHz, [D_6_]DMSO): *δ*=10.85 (br s, 1 H), 8.29 (br s, 2 H), 7.81 (t, *J*=1.7 Hz, 1 H), 7.71 (ddd, *J*=1.0, 2.0, 8.0 Hz, 1 H), 7.69–7.67 (m, 1 H), 7.63 (d, *J*=7.8 Hz, 2 H), 7.46 (t, *J*=7.8 Hz, 1 H), 7.39–7.36 (m, 2 H), 7.12–7.08 ppm (m, 1 H); LC–MS *m*/*z*=376 [*M*+H]^+^, *t*_R_=4.58 min, purity 100 % (Agilent, 20–90 % CH_3_CN, ES+ on an acidic method).

**(4-Amino-2-((2,6-dimethylphenyl)amino)thiazol-5-yl)(phenyl)methanone (17)**: General procedure A gave **17** as a yellow solid (35 mg, 0.11 mmol, 11 % yield); ^1^H NMR (500 MHz, [D_6_]DMSO): *δ*=10.11 (br s, 1 H), 8.40 (br s, 1 H), 7.97 (br s, 1 H), 7.54–7.16 (m, 8 H), 2.20 ppm (s, 6 H); LC–MS *m*/*z*=324 [*M*+H]^+^, *t*_R_=4.39 min, purity 95.6 % (Agilent, 20–90 % CH_3_CN, ES+ on an acidic method).

**(4-Amino-2-((3,4-dimethylphenyl)amino)thiazol-5-yl)(phenyl)methanone (18)**: General procedure A gave **18** as a yellow solid (100 mg, 0.30 mmol, 30 % yield); ^1^H NMR (500 MHz, [D_6_]DMSO): *δ*=10.60 (br s, 1 H), 8.31 (br s, 1 H), 8.11 (br s, 1 H), 7.67–7.65 (m, 2 H), 7.51–7.45 (m, 3 H), 7.34 (d, *J*=8.3 *Hz*, 1 H), 7.31 (s, 1 H), 7.12 (d, *J*=8.2 Hz, 1 H), 2.21 (s, 3 H), 2.19 ppm (s, 3 H); LC–MS *m*/*z*=324 [*M*+H]^+^, *t*_R_=4.55 min, purity 88 % (Agilent, 20–90 % CH_3_CN, ES+ on an acidic method); HRMS *m*/*z* [*M*+H]^+^ calcd for C_18_H_18_N_3_OS: 324.1165, found: 324.1158.

**(4-Amino-2-(cyclohexylamino)thiazol-5-yl)(phenyl)methanone (19)**: General procedure A gave **19** as a yellow solid (59 mg, 0.19 mmol, 19 % yield); ^1^H NMR (500 MHz, [D_6_]DMSO): *δ*=8.57 (br s, 1 H), 8.49 (br s, 1 H), 7.89 (br s, 1 H), 7.63–7.61 (m, 2 H), 7.47–7.42 (m, 3 H), 3.70 (br s, 1 H), 1.91 (d, *J*=10.7 Hz, 2 H), 1.76–1.70 (m, 2 H), 1.57 (d, *J*=12.9 Hz, 1 H), 1.32–1.13 ppm (m, 5 H); LC–MS *m*/*z*=302 [*M*+H]^+^, *t*_R_=4.42 min, purity 88 % (Agilent, 20–90 % CH_3_CN, ES+ on an acidic method); HRMS *m*/*z* [*M*+H]^+^ calcd for C_16_H_20_N_3_OS: 302.1322, found: 302.1321.

**(4-Amino-2-((cyclohexylmethyl)amino)thiazol-5-yl)(phenyl)methanone (20)**: General procedure A gave **20** as a yellow solid (136 mg, 0.43 mmol, 43 % yield); ^1^H NMR (500 MHz, [D_6_]DMSO): *δ*=8.63 (br s, 1 H), 8.43 (br s, 1 H), 7.84 (br s, 1 H), 7.63–7.61 (m, 2 H), 7.48–7.43 (m, 3 H), 3.17–3.03 (m, 2 H), 1.17–1.52 (m, 6 H), 1.23–1.11 (m, 3 H), 0.94–0.87 ppm (m, 2 H); LC–MS *m*/*z*=316 [*M*+H]^+^, *t*_R_=4.63 min, purity 97 % (Agilent, 20–90 % CH_3_CN, ES+ on an acidic method); HRMS *m*/*z* [*M*+H]^+^ calcd for C_17_H_22_N_3_OS: 316.1478, found: 316.1472.

**(4-Amino-2-(phenethylamino)thiazol-5-yl)(phenyl)methanone (21)**: General procedure A gave **21** as a yellow solid (77 mg, 0.24 mmol, 24 % yield); ^1^H NMR (500 MHz, [D_6_]DMSO): *δ*=8.73 (br s, 1 H), 8.47 (br s, 1 H), 7.87 (br s, 1 H), 7.63–7.61 (m, 2 H), 7.48–7.43 (m, 3 H), 7.32–7.29 (m, 2 H), 7.26–7.21 (m, 3 H), 2.90 (s, 1 H), 2.87 (t, *J*=7.2 Hz, 2 H), 2.74 ppm (d, *J*=0.5 Hz, 1 H); HRMS *m*/*z* [*M*+H]^+^ calcd for C_14_H_18_N_3_OS: 324.1165, found: 324.1153.

**1-(4-Amino-2-(cyclohexylamino)thiazol-5-yl)-2,2-dimethylpropan-1-one (22)**: General procedure A gave **22** as a yellow powder (171 mg, 0.61 mmol, 61 % yield); ^1^H NMR (500 MHz, CDCl_3_) *δ*=5.34 (d, *J*=7.1 Hz, 1 H), 3.30–3.28 (m, 1 H), 2.00 (dd, *J*=13.6, 3.3 Hz, 1 H), 1.70 (dt, *J*=13.6, 4.0 Hz, 2 H), 1.57 (dt, *J*=13.2, 4.0 Hz, 1 H), 1.38–1.33 (m, 2 H), 1.20 (s, 9 H, *t*Bu-H), 1.19–1.17 (m, 1 H), 0.81 (t, *J*=7.1 Hz, 1 H), 0.79–0.76 ppm (m, 1 H); ^13^C NMR (DMSO, 125 MHz) *δ*=128.6, 128.4, 119.5, 53.4, 53.3, 32.0, 27.0, 24.9, 24.3, 17.9, 16.6, 12.2 ppm; LC–MS *m*/*z*=282 [*M*+H]^+^, *t*_R_=4.60 min, purity 96 % (Agilent, 20–90 % CH_3_CN, ES+ on an acidic method); HRMS *m*/*z* [*M*+H]^+^ calcd for C_14_H_24_N_3_OS: 282.1635, found: 282.1641.

## Supplementary material

As a service to our authors and readers, this journal provides supporting information supplied by the authors. Such materials are peer reviewed and may be re-organized for online delivery, but are not copy-edited or typeset. Technical support issues arising from supporting information (other than missing files) should be addressed to the authors.
