# Supplementary material for: From On-Target to Off-Target Activity: Identification and Optimisation of Trypanosoma brucei GSK3 Inhibitors and Their Characterisation as Anti-Trypanosoma brucei Drug Discovery Lead Molecules
Source: ChemMedChem. 2013 Jun 14;8(7):1127–37. doi: 10.1002/cmdc.201300072 (PMC3728731; doi:10.1002/cmdc.201300072)
Supplement: Supplementary file 1 [file cmdc0008-1127-SD1.pdf]

CHEMMEDCHEM
Chemistry & Drug Discovery

Supporting Information

© Copyright Wiley-VCH Verlag GmbH & Co. KGaA, 69451 Weinheim, 2013

From On-Target to Off-Target Activity: Identification and Optimisation of *Trypanosoma brucei* GSK3 Inhibitors and Their Characterisation as Anti-*Trypanosoma brucei* Drug Discovery Lead Molecules

Andrew Woodland,[a] Raffaella Grimaldi,[a] Torsten Luksch,[a] Laura A. T. Cleghorn,[a] Kayode K. Ojo,[b] Wesley C. Van Voorhis,[b] Ruth Brenk,[a] Julie A. Frearson,[a] Ian H. Gilbert,*[a] and Paul G. Wyatt*[a]

cmdc_201300072_sm_miscellaneous_information.pdf

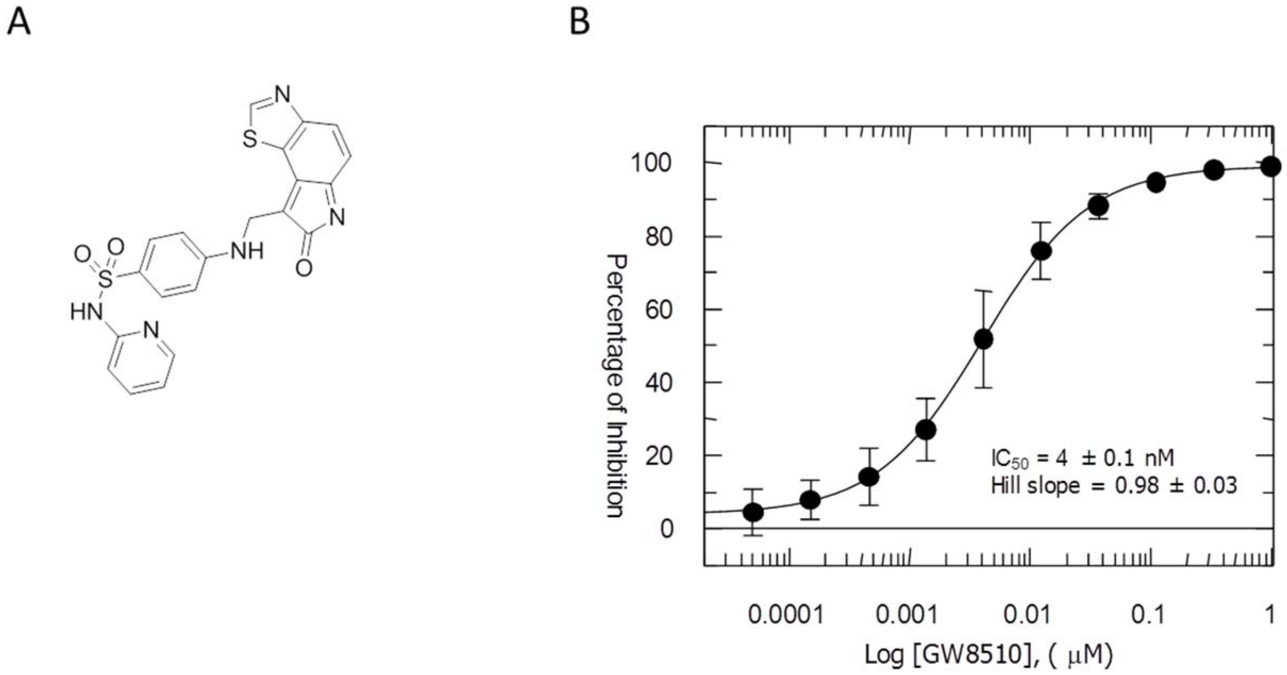

Figure S1: GW8510 Standard inhibitor IC_{50} determination against *T. brucei* GSK3. (**A**) Structure of GW8510 structure. (**B**). GW8510 prototypical inhibition curve (n=27 replicates). GW8510 was used as positive control in all assay plates and its potency monitored as measure of the assay reproducibility.

Table S1: Lists of compounds used to derive correlation plots (Figure 7 and Figure 9). Figure 7 was derived using the early compounds and Figure 9 was derived using both early and late examples of series 1.

	ID (Examples in paper numbered as per paper, examples not in paper numbered S"x")	Structure	Avg TbGSK3 IC50 (µmol)	count	Avg T.brucei EC50 (µmol)	count	logIC50	logEC50
early correlation	6	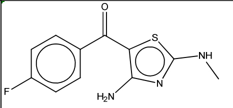	26	4	7.9	4	-4.6	-5.1
	S1	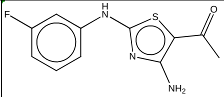	5.3	2	15	4	-5.3	-4.8
	7	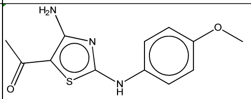	1.1	2	13	4	-6.0	-4.9
	1	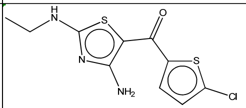	0.37	3	2.9	8	-6.4	-5.5
	S2	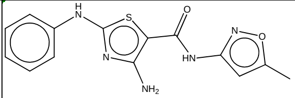	8	2	15	4	-5.1	-4.8
	S3	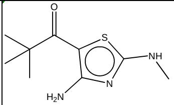	30	2	39	4	-4.5	-4.4
	S4	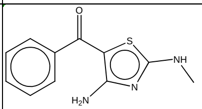	30	2	29	4	-4.5	-4.5
	S5	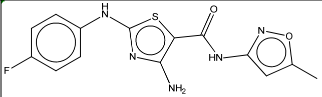	11	2	42	12	-5.0	-4.4

	ID (Examples in paper numbered as per paper, examples not in paper numbered S"x")	Structure	Avg TbGSK3 IC50 (µmol)	count	Avg T.brucei EC50 (µmol)	count	logIC50	logEC50
Early correlation	S6	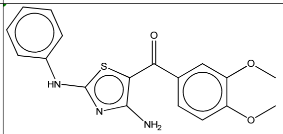	0.16	2	0.37	8	-6.8	-6.4
	8	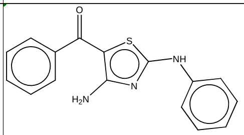	0.028	2	0.11	34	-7.6	-7.0
	S7	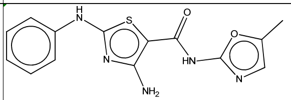	4.8	2	29	16	-5.3	-4.5
	9	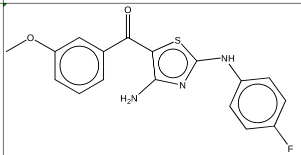	0.042	4	0.13	13	-7.4	-6.9
	S8	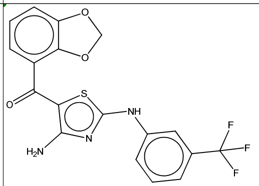	0.071	4	0.24	8	-7.1	-6.6
	S9	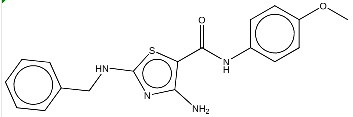	1	2	2.26	12	-6.0	-5.6
	10	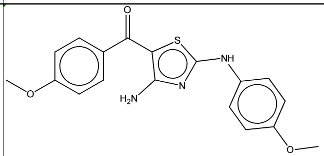	0.088	2	0.77	10	-7.1	-6.1

	ID (Examples in paper numbered as per paper, examples not in paper numbered S"x")	Structure	Avg TbGSK3 IC50 (µmol)	count	Avg T.brucei EC50 (µmol)	count	logIC50	logEC50
Early correlation	S10	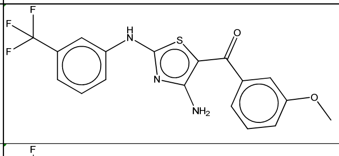	0.11	2	0.78	10	-7.0	-6.1
	S11	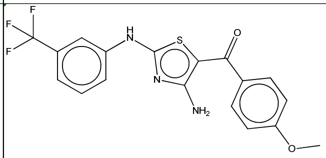	0.093	2	0.21	13	-7.0	-6.7
	S12	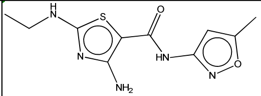	87	2	50	4	-4.1	-4.3
	S13	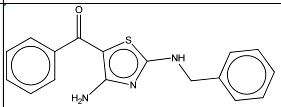	0.75	2	4.4	14	-6.1	-5.4
	S14	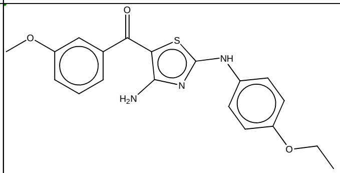	0.037	2	4	8	-7.4	-5.4
	S15	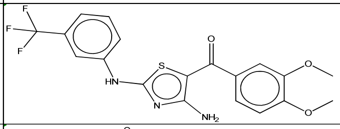	0.079	2	0.46	8	-7.1	-6.3
	S16	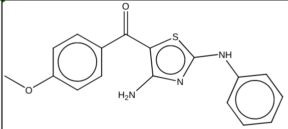	0.06	2	0.17	13	-7.2	-6.8

	ID (Examples in paper numbered as per paper, examples not in paper numbered S"x")	Structure	Avg TbGSK3 IC50 (µmol)	count	Avg T.brucei EC50 (µmol)	count	logIC50	logEC50
late correlation	23	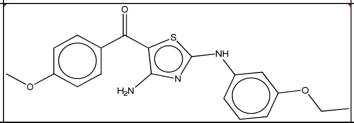	0.036	2	4.1	4	-7.4	-5.4
	15	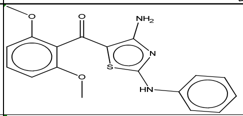	0.41	2	0.21	4	-6.4	-6.7
	16	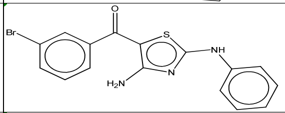	0.12	2	0.085	8	-6.9	-7.1
	S18	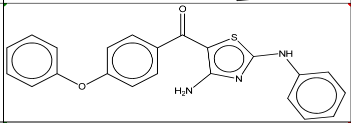	0.2	2	0.67	4	-6.7	-6.2
	S19	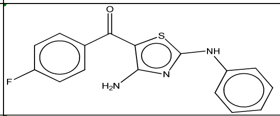	0.3	2	0.08	12	-6.5	-7.1
	17	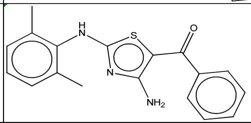	0.22	2	8.6	4	-6.7	-5.1
	S20	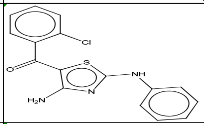	0.012	2	0.12	8	-7.9	-6.9
	S21	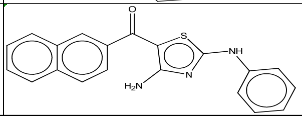	0.037	2	0.49	4	-7.4	-6.3

	ID (Examples in paper numbered as per paper, examples not in paper numbered S"x")	Structure	Avg TbGSK3 IC50 (µmol)	count	Avg T.brucei EC50 (µmol)	count	logIC50	logEC50
late correlation	21	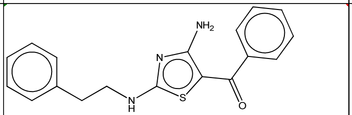	0.52	2	6.2	8	-6.3	-5.2
	S22	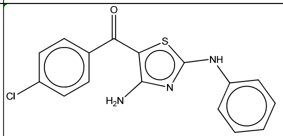	0.21	2	0.14	4	-6.7	-6.9
	20	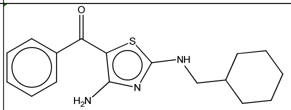	0.69	2	0.22	4	-6.2	-6.7
	18	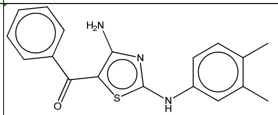	0.056	2	0.22	8	-7.3	-6.7
	19	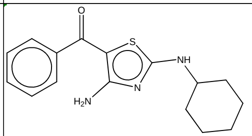	0.19	2	0.064	12	-6.7	-7.2
	S23	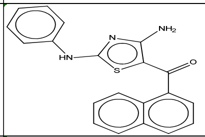	0.072	2	0.56	12	-7.1	-6.3
	14	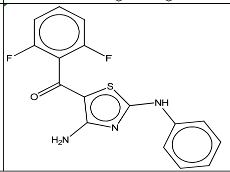	0.023	2	0.11	11	-7.6	-7.0

	ID (Examples in paper numbered as per paper, examples not in paper numbered S"x")	Structure	Avg TbGSK3 IC50 (µmol)	count	Avg T.brucei EC50 (µmol)	count	logIC50	logEC50
Late correlation	S24	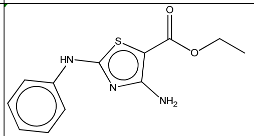	1.8	2	2.7	4	-5.7	-5.6
	S25	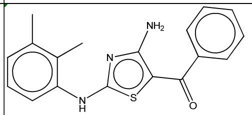	0.14	2	0.41	8	-6.9	-6.4
	S26	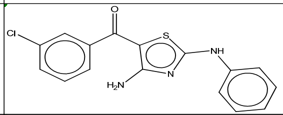	0.072	2	0.072	7	-7.1	-7.1
	S27	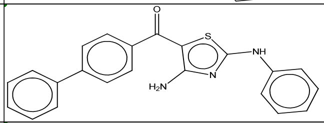	0.33	2	1.5	4	-6.5	-5.8
	22	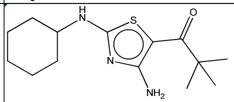	12		0.2		-4.9	-6.7

Table S2: Mammalian kinase selectivity panel (series 01). Compounds were tested in duplicate at 10 μM, inhibitor potency is expressed in terms of residual activity. The ranking of inhibition is shown as heat map: red>90% inhibition; orange 60-89% inhibition, yellow 30-59% inhibition; white below 30% of inhibition.

Enzyme	8	19	14	22
MKK1	0	68	96	94
ERK1	59	20	72	73
ERK2	26	16	68	95
JNK1	36	60	75	98
JNK2	48	39	81	90
p38α MAPK	14	24	8	102
P38β MAPK	47	22	12	82
p38γ MAPK	53	71	77	89
p38σ MAPK	56	67	62	102
ERK8	9	18	14	43
RSK1	24	67	29	89
RSK2	47	85	38	104
PDK1	64	106	65	108
PKBα	89	85	84	90
PKBβ	70	73	85	83
SGK1	80	81	98	91
S6K1	53	78	57	86
PKA	77	97	90	106
ROCK 2	42	97	55	107
PRK2	33	98	57	95
PKCα	70	101	64	100
PKC zeta	33	83	83	104
PKD1	29	35	46	85
MSK1	73	122	87	102
MNK1	71	105	110	105
MNK2	70	94	99	96
MAPKAP-K2	98	96	99	102
PRAK	72	96	63	103
CAMKKb	15	63	12	83
CAMK1	34	86	55	109
SmMLCK	22	44	29	74
PHK	74	105	88	112
CHK1	24	96	86	80
CHK2	27	61	30	53
GSK3β	0	1	4	15
CDK2-Cyclin A	2	5	2	13
PLK1	65	82	86	93
Aurora B	59	75	24	107

Enzyme	8	19	14	22
AMPK	41	96	67	101
MARK3	34	100	25	112
BRSK2	42	93	55	95
MELK	34	70	47	89
CK1	14	82	13	74
CK2	107	114	111	118
DYRK1A	12	25	10	88
DYRK2	25	39	79	90
DYRK3	51	57	83	96
NEK2a	68	93	92	93
NEK6	60	113	97	100
IKKb	55	70	82	99
PIM1	71	94	79	90
PIM2	80	94	91	109
PIM3	32	69	69	82
SRPK1	26	93	6	92
MST2	42	97	40	104
EF2K	84	89	95	111
HIPK2	3	64	16	79
PAK4	32	88	47	88
PAK5	38	103	66	110
PAK6	52	93	87	98
SRC	42	99	47	99
LCK	53	86	49	85
CSK	57	95	90	98
FGF-R1	39	97	64	87
IRR	16	25	24	93
EPH A2	77	110	71	106
MST4	65	76	57	90
SYK	45	91	37	111
YES1	25	114	26	109
IKKε	35	88	84	108
TBK1	53	88	92	95
IGF-1R	41	80	80	118
VEG-FR	30	57	17	82
BTK	60	75	68	79
IR-HIS	63	75	88	93
EPH-B3	78	88	59	94
